# SAFETY AND EFFECTIVENESS OF SINGLE ANASTOMOSIS DUODENAL SWITCH PROCEDURE: PRELIMINARY RESULT FROM A SINGLE INSTITUTION

**DOI:** 10.1590/0102-6720201600S10020

**Published:** 2016

**Authors:** Lars NELSON, Rena C. MOON, Andre F. TEIXEIRA, Manoel GALVÃO, Almino RAMOS, Muhammad A. JAWAD

**Affiliations:** 1 Department of Bariatric Surgery, Orlando Regional Medical Center, Orlando Health, Orlando, FL, USA; 2 Gastro Obeso Center, São Paulo, Brazil

**Keywords:** Single anastomosis, Duodeno-ileal bypass, Safety, Efficacy

## Abstract

**Background:**

Single anastomosis duodeno-ileal bypass with sleeve gastrectomy (SADI-S) was introduced into bariatric surgery by Sanchez-Pernaute et al. as an advancement of the biliopancreatic diversion with duodenal switch.

**Aim:**

To evaluate the SADI-S procedure with regard to weight loss, comorbidity resolution, and complication rate in the super obese population.

**Methods:**

A retrospective chart review was performed on initial 72 patients who underwent laparoscopic or robot-assisted laparoscopic SADI-S between December 17^th^, 2013 and July 29^th^, 2015.

**Results:**

A total of 48 female and 21 male patients were included with a mean age of 42.4±10.0 years (range, 22-67). The mean body mass index (BMI) at the time of procedure was 58.4±8.3 kg/m^2^ (range, 42.3-91.8). Mean length of hospital stay was 4.3±2.6 days (range, 3-24). Thirty-day readmission rate was 4.3% (n=3), due to tachycardia (n=1), deep venous thrombosis (n=1), and viral gastroenteritis (n=1). Thirty-day reoperation rate was 5.8% (n=4) for perforation of the small bowel (n=1), leakage (n=1), duodenal stump leakage (n=1), and diagnostic laparoscopy (n=1). Percentage of excess weight loss (%EWL) was 28.5±8.8 % (range, 13.3-45.0) at three months (n=28), 41.7±11.1 % (range, 19.6-69.6) at six months (n=50), and 61.6±12.0 % (range, 40.1-91.2) at 12 months (n=23) after the procedure. A total of 18 patients (26.1%) presented with type II diabetes mellitus at the time of surgery. Of these patients, 9 (50.0%) had their diabetes resolved, and six (33.3%) had it improved by 6-12 months after SADI-S.

**Conclusions:**

SADI-S is a feasible operation with a promising weight loss and diabetes resolution in the super-obese population.

## RESUMO

**Racional:**

Anastomose única em bypass duodenoileal com gastrectomia vertical (SADI-S) foi introduzida na cirurgia bariátrica por Sanchez-Pernaute et al. como um avanço da derivação biliopancreática com switch duodenal.

**Objetivo:**

Avaliar o procedimento SADI-S no que diz respeito à perda de peso, resolução de comorbidades e taxa de complicações na população de superobesos.

**Métodos:**

Estudo retrospectivo com 72 pacientes iniciais que foram submetidos à laparoscopia ou por robô-assistida SADI-S laparoscópica entre 17 de dezembro de 2013 e 29 de Julho de 2015.

**Resultados:**

Foram incluídos 48 pacientes do sexo feminino e 21 do masculino com média idade de 42,4±10,0 anos (variação, 22-67). O índice de massa corporal (IMC) no momento do procedimento foi de 58,4±8,3 kg/m2 (42,3-91,8). O tempo médio de permanência hospitalar foi de 4,3±2,6 dias (3-24). A taxa de readmissão em 30 dias foi de 4,3% (n=3), devido à taquicardia (n=1), trombose venosa profunda (n=1), e gastroenterite viral (n=1). A taxa de reoperação em 30 dias foi de 5,8% (n=4) para a perfuração do intestino delgado (n=1), fístula (n=1), deiscência do coto duodenal (n=1), e laparoscopia de diagnóstico (n=1). Percentagem de excesso de perda de peso (% PEP) foi de 28,5±8,8% (13,3-45,0) em três meses (n=28), 41,7±11,1% (19,6-69,6) em seis meses (n=50), e 61,6±12,0% (40,1-91,2) aos 12 meses (n=23) após o procedimento. Um total de 18 pacientes (26,1%) apresentou-se com diabete melito tipo 2, no momento da operação. Desses, nove (50,0%) tiveram seu diabete resolvido, e seis (33,3%) tinham melhorado em 6-12 meses após SADI-S.

**Conclusões:**

SADI-S é operação viável com promissora perda de peso e de resolução do diabete melito na população de superobesos.

## INTRODUCTION

The first bariatric procedure was the jejunocolic bypass followed by the jejunoileal bypass, which resulted in substantial weight loss but unacceptable life threatening complication rates. These procedures along with several others have fallen out of favor over the years, due to failure rates, health risks, and severe deficiencies^3^ ^,^ ^4^. Currently, laparoscopic Roux-en-Y gastric bypass (RYGB) and laparoscopic sleeve gastrectomy (LSG) are most commonly performed for surgical treatment of morbid obesity^1^.

Surgery for the super obese (body mass index >50 kg/m^2^) carries higher readmission and reoperation rates^19^. The main objective of bariatric operations is maximizing weight loss, while maintaining or achieving nutritional equilibrium, and preventing micronutrient and protein loss^6^. Biliopancreatic bypass was first described by Scopinaro in 1979^17^. The classic construction is a laparoscopic or robotic technique combining sleeve gastrectomy with creating a 100 cm common channel with a 200 cm alimentary limb in two anastomosis, Roux-en-Y configuration^2^ ^,^ ^11^. This procedure has a steep learning curve and some reports at least 50 procedures before proficiency is obtained^11^ ^,^ ^21^. 

Single anastomosis duodenoileal bypass with sleeve gastrectomy (SADI-S) was introduced into bariatric surgery by Sanchez-Pernaute et al.^14^ as an advancement of the biliopancreatic diversion with duodenal switch. Since then, the group has modified and improved their technique for optimal results^13^ ^,^ ^15^ ^,^ ^16^. 

This study aims to evaluate the single anastomosis (SADI-S) procedure with regard to weight loss, comorbidity resolution, and complication rate in the super obese population, and will be the first report of the technique in the United States. 

## METHODS

After institutional review board approval and following the Health Insurance Portability and Accountability Act guidelines, the authors performed a retrospective chart review of a prospectively maintained database of initial 72 patients who underwent laparoscopic or robot-assisted laparoscopic SADI-S between December 17^th^, 2013 and July 29^th^, 2015. For this type of study, formal consent is not required.

SADI-S was performed by two surgeons according to the National Institutes of Health criteria for the management of morbid obesity. Patients were followed up at our office clinic at 1, 3, 6, 12 months postoperatively and yearly thereafter. Follow-up visits included weight measurement, clinical history and examination, and laboratory tests for blood glucose as well as nutrition deficiency. Comorbid conditions were recorded at each visit. Remission of hypertension (HTN) was defined as blood pressure below 140/90 mmHg without medication. Remission of diabetes mellitus (DM) was defined as fasting glucose level below 100 mg/dl without medication. Improvement of DM was defined as fasting glucose level below 100 mg/dl decrease in number or dosage of medication. Remission of sleep apnea was based on patient's statement and no usage of continuous positive airway pressure machine. 

All data for age and BMI are demonstrated as mean±standard deviation, unless otherwise noted. Statistical analysis was performed using descriptive analysis and two-tailed Student's t-test, with p<0.05 regarded as statistically significant. 

### Surgical Technique

**FIGURE 1:**
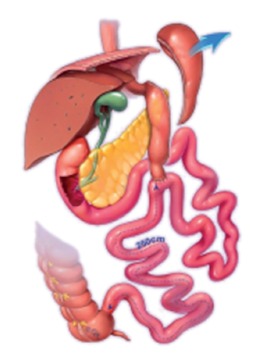
Diagram of the single anastomosis duodenoileal bypass with sleeve gastrectomy

**FIGURE 2:**
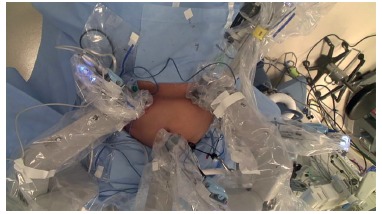
Positioning of trocars after docking the robot

**FIGURE 3:**
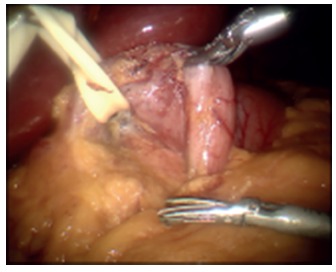
Placement of the Penrose drain at the duodenum

**FIGURE 4:**
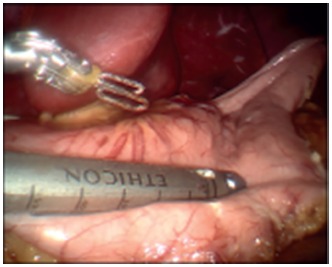
Creation of sleeve over a 34-French bougie-sized Edlich tube with a linear stapler

**FIGURE 5:**
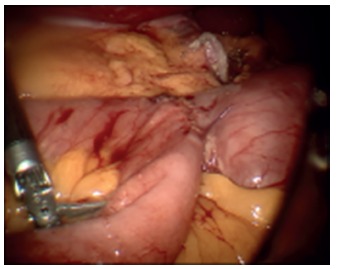
Creation of hand-sewn duodenoileal anastomosis

All procedures, except 10 initial cases, were performed using the da Vinci-assisted robotic system (Intuitive Surgical, Sunnyvale, CA, USA). None of the cases were converted to open. The left lobe of the liver was retracted anteriorly. The terminal ileum was identified and run for 250 cm proximal and marked with a two sutures (Figure 1). The da Vinci robot was docked (Figure 2). The inferior border of the distal antrum and the proximal duodenum was dissected robotically creating a window underneath the duodenum which was isolated with a Penrose drain (Figure 3). The greater omentum was detached from the greater curvature of the stomach 6 cm from the pylorus all the way up to the duodenojejunal angle. The greater curve of the stomach was transected over a 34 French bougie-sized Edlich tube (Covidien, Mansfield, MA, USA) with a linear stapler creating the sleeve (Figure 4). Next, the duodenum was transected with a linear stapler through the previously created window. After that, the marked ileum was brought up to the proximal duodenum and a 2-layer hand-sewn duodenoileal anastomosis was created (Figure 5). The anastomoses and sleeve were tested with methylene blue and air to evaluate for a leak. A drain was placed in the left upper quadrant across the anastomosis. Twenty-six (36.1%) patients also underwent cholecystectomy at the time of SADI-S procedure. 

## RESULTS

Out of 72 patients, three underwent the procedure as a two-step, and were excluded from the study. A total of 48 female and 21 male were included with a mean age of 42.4±10.0 years (range, 22-67). The mean body mass index (BMI) at the time of procedure was 58.4±8.3 kg/m^2^ (range, 42.3-91.8). Demographics of the patients are listed in Table 1.

**TABLE 1:** Demographics of single anastomosis duodenoileal bypass patients

Number of patients Female Male	69 48 (69.6%) 21 (30.4%)
Age (years)^a^	42.4±10.1 (range 22-67)
Body Mass Index (kg/m^2^)^a^	58.4±8.3 (range 42.3-91.8)
Comorbidities Hypertension Diabetes mellitus Sleep apnea	33 (47.8%) 18 (26.1%) 20 (29.0%)

^a^ At the time of procedure

### Complications

Mean length of hospital stay was 4.3±2.6 days (range, 3-24). Six patients had a prolonged hospital stay (longer than five days) due to decreased oral intake (n=3), atelectasis (n=1), postoperative bleeding (n=1), and duodenoileal obstruction with perforation of the small bowel (n=1). The patient with obstruction was taken to the operating room (OR) on postoperative day (POD) 7 for relief of obstruction, lysis of adhesions and repair of the enterotomies. 

Thirty-day readmission rate was 4.3% (n=3), due to tachycardia (n=1), deep venous thrombosis (n=1), and viral gastroenteritis (n=1). Thirty-day reoperation rate was 5.8% (n=4) for perforation of the small bowel (n=1), leakage (n=1), duodenal stump leakage (n=1), and diagnostic laparoscopy (n=1). Patient with a leakage developed pain and tachycardia, and was diagnosed with a leak and peritonitis on POD 1 before discharge. She was taken back to the OR and underwent en-bloc resection with conversion to a loop gastrojejunostomy and drain placement (mini-gastric bypass). She did well after the reoperation and was discharged on POD 5. Another patient presented to the emergency department on POD 11 with nausea, fatigue, and severe abdominal pain. Exploratory laparotomy revealed hemoperitoneum and duodenal stump blowout, which was oversewn^9^.

### Weight loss and comorbidities

 One patient had less than 30-days of follow-up and therefore excluded from the data analysis. In 68 patients, mean follow-up period was 9.5±5.9 months (range, 1-25).

Percentage of excess weight loss (%EWL) was 28.5±8.8 % (range, 13.3-45.0) at three months (n=28), 41.7±11.1% (range, 19.6-69.6) at six months (n=50), and 61.6±12.0% (range, 40.1-91.2) at 12 months (n=24) after the procedure (Table 2). 

**TABLE 2:** Weight reduction and change in comorbidities in single anastomosis duodenoileal bypass patients

	Single anastomosis duodenoileal bypass (n=69)							
	n	%EWL	%EBMIL	%WL		HTN	DM	OSA
3 mo	28	28.5	30.8	17.3	Preoperative	33	18	20
6 mo	50	41.7	45.3	25.2	Improvement	8 (24.2%)	6 (33.3%)	N/A
12 mo	24	61.6	66.7	37.3	Remission	14 (42.4%)	9 (50.0%)	12 (60.0%)

* One patients had less than 30-day of follow-up; *n in each procedure shows the number of patients available with weight information at each check point; %EWL=percentage of excess weight loss; %EBMIL =percentage of excess body mass index loss; % WL=percentage of weight loss; mo=months; HTN=hypertension; DM=diabetes mellitus; OSA=obstructive sleep apnea

### Comorbidities

A total of 18 patients (26.1%) presented with type II DM at the time of surgery. Of these, 9 (50.0%) had their DM resolved, and six (33.3%) improved by six months after SADI-S. Two patients had not followed-up longer than three months and one remained having DM with the same medication at one year follow-up. One patient who had fasting blood glucose level of 338 mg/dl and HbA1c of 14% preoperatively showed a dramatic decrease of fasting blood glucose level of 79 mg/dl without medication at 6-month follow-up (Table 2). 

Out of 33 patients (47.8%) with HTN at the time of surgery, 14 (42.4%) had their HTN resolved at six months after SADI-S. Out of 20 (29.0%) with obstructive sleep apnea, 12 (60.0%) had it resolved at six months after the procedure. 

### Laboratory results

In 24 patients with longer than one-year follow-up, laboratory results were analyzed at one-year checkpoint (Table 3). Six (25.0%) had low hemoglobin levels and four (16.6%) had low hematocrit without presenting clinical symptoms. These patients were instructed to adamantly take or increase their iron intake. Two to three patients had hypoproteinemia and hypoalbuminemia, however, did not complain of clinical symptoms. The most deficient element was vitamin D at 45.8% (n=11). These patients were instructed to take higher doses of vitamin D_3_.

**TABLE 3:** Postoperative laboratory results after single anastomosis duodenoileal bypass in 24 patients with one-year follow-up

	Mean at one-year follow-up	Number of patients with abnormal value
Hemoglobin (g/dl)	13.2±1.9 (range, 10.3-18.6)	6 (25%)
Hematocrit (%)	40.2±5.5 (range, 29.4-55.8)	4 (17%)
Proteins (g/dl)	6.7±0.5 (range, 5.7-7.9)	2 (8%)
Albumin (g/dl)	3.9±0.5 (range, 2.6-5.0)	3 (13%)
Vitamin B12 (pg/ml)	940.7±522.4 (range, 280-2000)	0
Calcium (mg/dl)	9.0±1.0 (range, 5.2-10.2)	3 (13%)
Vitamin D (ng/ml)	29.7±14.3 (range, 7-63)	11 (46%)

## DISCUSSION

The duodenal switch procedure has a profound impact on the obesity and ample literature exists comparing it to other bariatric procedures^8^ ^,^ ^10^ ^,^ ^12^ ^,^ ^14^ ^,^ ^20^. Furthermore, the duodenal switch has shown more rapid sustainable weight loss and comorbidity resolution when compared to these other procedures especially in the super-obese patient population^8^ ^,^ ^10^ ^,^ ^12^. This procedure combines restriction, malabsorption, and hormonal changes to achieve weight loss and comorbidity resolution^18^. Several iterations of duodenal switch exist and it has evolved over time. Scopinaro et al.^17^ pioneered the way with his initial experience in humans by performing a distal gastrectomy and a long Roux-en-Y construction with a gastroileal anastomosis. Two more recent alterations of the procedure exist including the traditional duodenal switch with a vertical sleeve gastrectomy and a Roux-en-Y duodenoileal construction, and a duodenal switch consisting of a sleeve gastrectomy with a single anastomosis duodenoileal billroth II construction (SADI-S). 

 Sanchez-Pernaute et al.^14^ first described SADI-S in hopes of simplifying the duodenal switch procedure. The goal of this approach was to reduce the number of intestinal anastomoses, postoperative leaks, obstructions or anastomotic strictures, operative time and anesthetic-related complications. They reported an excellent weight loss and comorbidities resolution with minimal complications at three years of follow-up^13^. They initially chose the efferent loop length of 200 cm, but lengthened it to 250 cm due to hypoalbuminemia and excessive malabsorption issues^16^. They did not find a significant difference in weight loss and comorbidity resolution between the two different efferent loop length, and almost 100% of their patients maintained more than 99% of %EWL^16^. 

We adopted this procedure in December 2013, with the efferent loop length of 250 cm. For the sleeve, we used a 34 French-sized bougie and made the sleeve very loose against the tube. Our choice of patients erred on the heavier side, making the mean preoperative BMI 58 kg/m^2^. Our patients showed %EWL of 42% and 62% at six and 12 months, respectively. While the weight loss was significant (great than >50% of %EWL at one year) considering these patients had a high BMI to begin with, we were not able to reproduce 95% of %EWL at 12 months as Sanchez-Pernaute et al.^16^ have reported. Many studies have found that patients with a BMI >60 kg/m^2^ experience less weight loss than those with a lower BMI after gastric bypass^22^ ^,^ ^23^. We also recently reported that BMI 40-50 kg/m^2^ group lost more than the BMI 50-60 kg/m^2^ group, and the BMI 50-60 kg/m^2^ group lost more than the BMI ≥60 kg/m^2^ group after gastric bypass^7^. This may be the reason for discrepancy in %EWL as our patients had a mean BMI of 58 kg/m^2^ while their patients had a mean BMI of 45 kg/m^2^. This may be because the first 50 patients included in his data set had a shorter common channel of 200 cm skewing the data toward a higher and more sustainable %EWL compared with our common channel of 250 cm. Further, other factors such as variation in post-operative practices and bariatric protocols such as dieting may influence the rate and extent of weight loss. They maintained patients on a low caloric diet for the first postoperative month. It is our practice to encourage higher caloric intake as tolerated while being maintained on a phase 2 diet.

Marceau et al.^5^ analyzed their result following a traditional duodenal switch. They further stratified their patient population into a super obese group with BMI >50 kg/m^2^. They defined success as "cure rate" as achieving a BMI <40 kg/m^2^ and their cure rate was 83% for the super obese group. Using this definition, 72% of our patients with available one-year follow-up achieved BMI <40 kg/m^2^ at one year.

SADI-S is a malabsorptive and restrictive procedure inducing weight loss and decreasing absorption of vital nutrients. Twenty-five percent of the patients had abnormally low values of hemoglobin or hematocrit. These values improved with increased oral iron intake and did not require transfusion. Eight percent (n=2) and 13% (n=3) of the patients had below normal limits mean total protein and albumin concentrations, respectively. 

The duodenal switch has shown excellent comorbidity resolution profile. Sanchez-Pernaute et al.^14^ had 100% resolution in DM. In our study, 26% presented with type II DM at the time of surgery, and 50% of these had their DM resolved. Higher BMI and slower weight loss may have resulted in fewer patients with DM resolution. Also, our results may be more comparable if we had a longer follow up.

Our reoperation rate was 5.8% (n=4), including one leak at the duodenoileal anastomosis and another at the duodenal stump. Similar to our series, Sanchez-Pernaute et al.^16^ experienced a low reoperation and leak rate. They reported three leaks in their first 100 cases, one from the duodenoileal anastomosis and the other two from the sleeve gastrectomy staple line. Unlike our series, all of theirs were treated conservatively. 

In accordance with current literature, decreased levels of vitamins and micronutrients were observed in our study. These abnormalities were not severe and improved with increased oral supplementation. However, we should note the high rate (46%) of vitamin D deficiency in our patients. Due to risk of osteoporosis, we recently employed a routine bone scan after one year following SADI-S. 

This study has several limitations. One is the small sample size leading to the possibility that our data for SADI-S is under powered leading to less accuracy. Secondly, this study is a retrospective review. A prospective randomized control trial may better assess patient outcomes and comorbidity resolution. Third, this study shows short term follow-up on our initial experience with SADI-S. Long term data would help confirm or refute how truly robust this procedure is and may show a better side effect profile. 

## CONCLUSIONS

SADI-S is a feasible operation with a promising weight loss and diabetes resolution in the super-obese population. 
